# APLP1 is endoproteolytically cleaved by γ-secretase without previous ectodomain shedding

**DOI:** 10.1038/s41598-018-19530-8

**Published:** 2018-01-30

**Authors:** Linda Schauenburg, Filip Liebsch, Murat Eravci, Magnus C. Mayer, Christoph Weise, Gerhard Multhaup

**Affiliations:** 1 Institut für Chemie und Biochemie, Freie Universität Berlin, Thielallee 63, 14195 Berlin, Germany; 2 Department of Pharmacology & Therapeutics and Integrated Program in Neuroscience, McGill University, 3655 Promenade Sir William Osler, Montreal, QC H3G 1Y6 Canada; 3 Present Address: Sphingotec Therapeutics GmbH, Neuendorfstr. 15a, 16761 Hennigsdorf, Germany; 4 Present Address: Miltenyi Biotec GmbH, Robert-Koch-Strasse 1, 17166 Teterow, Germany

## Abstract

Regulated intramembrane proteolysis of the amyloid precursor protein (APP) and its homologs, the APP like proteins APLP1 and APLP2, is typically a two-step process, which is initiated by ectodomain-shedding of the substrates by α- or β-secretases. Growing evidence, however, indicates that the cleavage process for APLP1 is different than for APP. Here, we describe that full-length APLP1, but not APP or APLP2, is uniquely cleaved by γ-secretase without previous ectodomain shedding. The new fragment, termed sAPLP1γ, was exclusively associated with APLP1, not APP, APLP2. We provide an exact molecular analysis showing that sAPLP1γ was uniquely generated by γ-secretase from full-length APLP1. Mass spectrometry analysis showed that the sAPLP1γ fragment and the longest Aβ-like peptide share the C-terminus. This novel mechanism of γ-secretase action is consistent with an ϵ-cut based upon the nature of the reaction in APP. We further demonstrate that the APLP1 transmembrane sequence is the critical determinant for γ-shedding and release of full-length APLP1. Moreover, the APLP1 TMS is sufficient to convert larger type-I membrane proteins like APP into direct γ-secretase substrates. Taken together, the direct cleavage of APLP1 is a novel feature of the γ-secretase prompting a re-thinking of γ-secretase activity modulation as a therapeutic strategy for Alzheimer disease.

## Introduction

APLP1 (amyloid precursor-like protein 1) is part of an evolutionarily conserved family of type-I transmembrane proteins including the amyloid precursor protein (APP) and APLP2 (amyloid precursor-like protein 2) which are homologous in function^1–4^. APP and APLPs have roles in neuronal differentiation, synaptogenesis, neurite outgrowth, and synaptic plasticity^5–12^. These proteins are expressed in different tissues and cell types. They bind to components of the extracellular matrix^8,13,14^, help mediate cell–cell interactions, and form homo- and heterotypic protein interactions in a modular mode^15–17^.

APLP1 is an atypical member of the APP family. APLP1 expression is neuron-specific^18,19^, and its subcellular localization and oligomerization properties are different from those of APP and APLP2^15^. APP and APLPs are substrates for neuronal adhesion with a purely zinc-dependent adhesive function for APLP1^20^. As a neuronal adhesion receptor, APLP1 supports maintenance of dendritic spines and basal synaptic transmission while APP and APLP2 exhibit a basal adhesive activity. Zinc induces formation of neuronal contacts, primarily mediated by APLP1 high-order oligomers^21^. Silencing of APLP2 revealed a function in neurogenesis in an APP/APLP1 double knockout mouse background implying that APLP2 has a distinct role in priming cortical progenitors for neuronal differentiation^22^.

Shedding of APP and APLPs by α- or β-secretases (BACE1) results in the generation of membrane-bound C-terminal fragments (CTF) and soluble ectodomains that were recently detected as APP heteromers in human CSF^23^. APP processing by BACE1 appears to be tightly regulated by BACE1 expression. In contrast, APLP1 and 2 are less tightly regulated and depend less on BACE1 expression^24^. The production of intracellular domains (ICDs) for all three family members is not reduced with decreased BACE1 activity^24^, a finding that has yet to be explained. Subsequent γ-cleavage of membrane-bound stubs of APLP1 (i.e. products of α- or β-cuts) generate Aβ-like peptides with 25, 27 and 28 residues (denoted APL1β25, 27, 28). APL1β28 has been suggested to be a surrogate marker for Aβ1–42 production in the brain^25^. Peptides APL1β25, 27 and 28 were detected in human CSF and found to be decreased in Down syndrome (DS) patients compared to controls without any correlation to age or sex^26^. Since APL1β28 was found to be increased in CSF of AD patients^25^, this may indicate altered γ-secretase cleavage activity in AD pathogenesis.

The enzymes involved in APLP1 ectodomain processing are less well defined^27^. Shedding of APP and APLPs by the α- and β-secretases results in the generation of membrane-bound CTFs. CTFs are further processed into ICDs which are postulated to be involved in transcriptional regulation^28–30^. Clustering of APP and APLPs impair their normal processing by secretases leading to reduced levels of soluble APPs and APLPs^16^. Zinc enriches APP family members at sites of cell-cell contacts and revealed a zinc-dependent role of APP/APLPs as neuronal cell adhesion proteins^21^.

Furthermore, the release of full-length APLP1^24^, APLP1-derived ICD (AL1ICD) production, and cellular localization of APLP1 indicated a slightly different cleavage process and a specific cytosolic role of AL1ICD in the regulation of transcription in comparison to its APP and APLP2 counterparts^31^. However, the sequence of AL1ICD is not well defined since the ICD γ-secretase cleavage site is rather based on alignments and on indirect analysis by Western blot than on an exact molecular analysis^24,31,32^.

Here, we describe that APLP1, but not APP or APLP2, is cleaved by γ-secretase without previous ectodomain shedding. The new fragment, termed sAPLP1γ, was exclusively associated with APLP1 in the absence and presence of α-/β-secretase inhibitors, but not with APP or APLP2 or when γ-secretase activity was inhibited. The sAPLP1γ fragment was uniquely generated by γ-secretase from full-length APLP1, mainly in a competing reaction with BACE1 and detected as a released form of APLP1-overexpressing cells (HEK293T and SH-SY5Y) but also wild-type SH-SY5Y cells. Our sequence analysis showed that sAPLP1γ and the APL1β28 peptide share the same C-terminus. Mutational analyses revealed that the critical and decisive factor for γ-shedding and release of full-length APLP1 from the membrane is the APLP1 transmembrane sequence (TMS). The APLP1 TMS is sufficient to convert the large type-I membrane protein APP into a direct γ-secretase substrate.

## Results

### APLP1, but not APP or APLP2, is cleaved by γ-secretase without previous ectodomain shedding

While BACE1 has a key role in the regulation of APLP1 maturation, trafficking, and secretion^33^, the loss of BACE1 processing of APLP1 has no detrimental effect on the *de novo* production of AL1ICD^24,31^. In the present study, we wanted to elucidate whether there is a γ-secretase-mediated cleavage of APLP1 without previous α- or β-processing. If true, this would ensure a certain cellular level of AL1ICD generated from APLP1 in a single-stage process.

**Figure 1:**
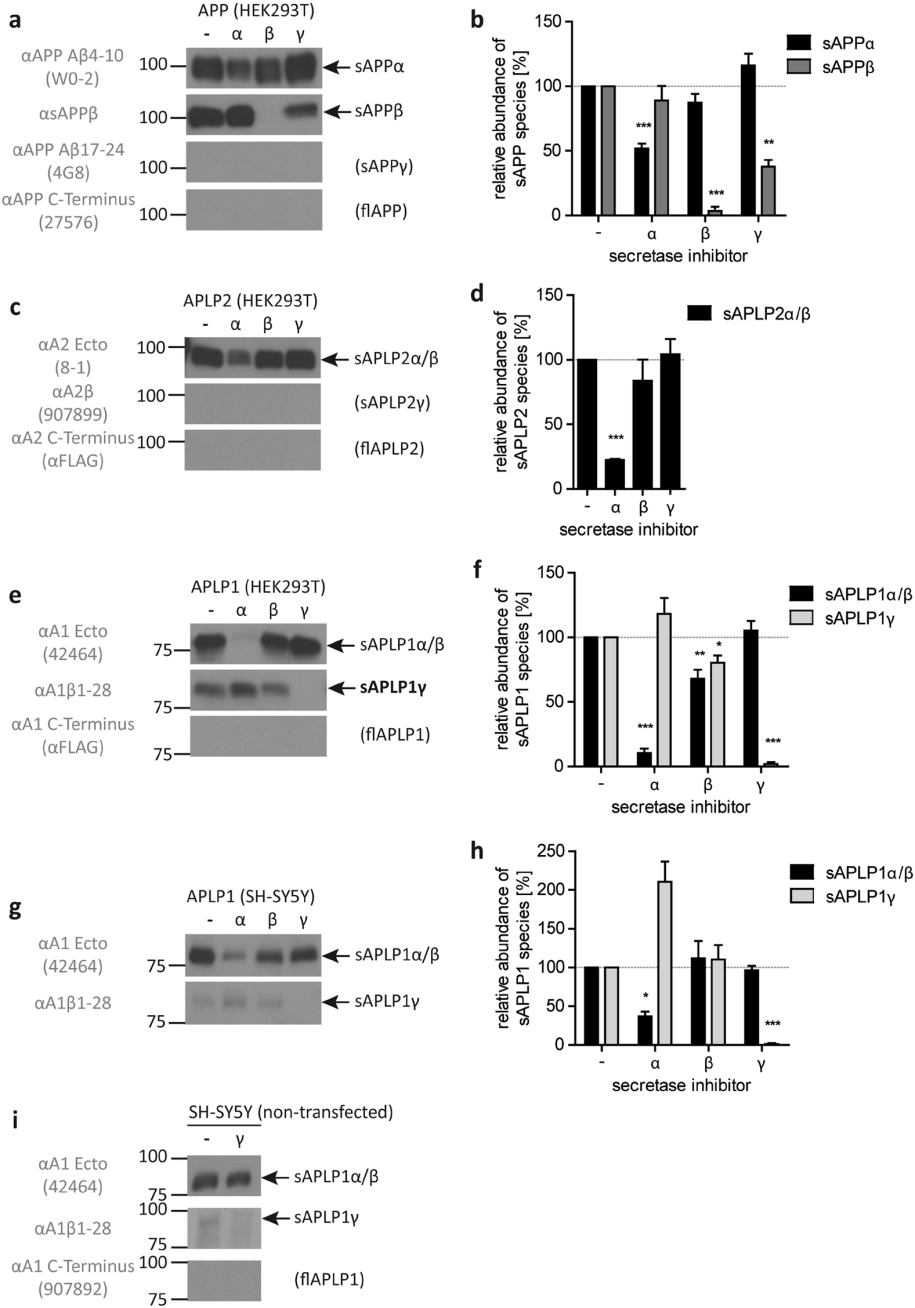
Western blot analysis of APP, APLP2 and APLP1 processing in HEK293T and SH-SY5Y cells and quantitative analyses of detected fragments. Western blot analysis of soluble fragments in the media of HEK293T cells overexpressing APP (**a**), APLP2-FLAG (**c**) and APLP1-FLAG (**e**) as well as APLP1-overexpressing SH-SY5Y cells (**g**) and of non-transfected SH-SY5Y cells with or without γ-secretase inhibition after immunoprecipitation from conditioned medium with αAPLP1ecto antibody and detection with the antibodies 42464, αA1β1–28 and 907892 (**i**). Conditioned media were analyzed using specific antibodies for the soluble ectodomains (APLP1–42464; APLP2–8–1; APP – W0–2 or αsAPPβ), the Aβ-region (APLP1 – αA1β1–28; APLP2–907899; APP – 4G8) or the C-terminus (APLP1/APLP2 – αFLAG; APP – 27576). Transfected cells were treated with α- (GM6001, Calbiochem), β- (β-secretase inhibitor IV, Calbiochem) or γ-secretase inhibitor (L-685,458 Calbiochem). All respective bands were quantified, normalized against the untreated control (100%) and statistically analyzed by one-sample t-test vs. 100% with Bonferroni correction (*p < 0.05, **p < 0.01, ***p < 0.001, (**b**,**d**,**f**,**h**)). Displayed are representative Western blots of at least three independent experiments. The actual number of repeats and representative full-length blots are given and presented in Supplementary Figure 1, respectively.

To confirm the preceding observation that soluble forms of APP and APLPs are generated by specific secretases^34^, we analyzed soluble fragments in the conditioned media of HEK293T cells transiently overexpressing either APP, APLP1 or APLP2 with and without the respective secretase inhibitor (Fig. 1a–f). Soluble fragments were detected with specific antibodies, as described in the experimental procedures (see Methods section for antibodies). The level of soluble α-secretase-cleaved APP (sAPPα) was significantly reduced upon α-secretase inhibition by GM6001 treatment, which is a broad-spectrum matrix-metalloprotease (MMP) and α-secretase (a disintegrin and metalloproteinase, ADAM10) inhibitor^35^. The more specific BACE1 inhibitor IV completely abolished the detection of BACE1-mediated soluble APP (sAPPβ) secretion while the γ-secretase inhibitor L-685,458 had no effect on either sAPP form (Fig. 1a,b). To address the potential production and release of C-terminally longer cleavage products than sAPPα, we used a monoclonal antibody recognizing Aβ residues 17–24 and a polyclonal antibody directed against the C-terminal APP domain^36^. Both antibodies failed to detect full-length APP or other C-terminally truncated forms that would carry the respective epitopes and indicated its absence in the supernatant (Fig. 1a). Furthermore, polyclonal antibodies against APLP2 raised against the Aβ-like region A2β and the cytoplasmic domain or the C-terminal FLAG tag failed to detect the respective proteins. These results indicate the absence of full-length APLP2 or C-terminally extended forms of soluble α/β-cleaved domains (sAPLP2α/β) in the supernatant (Fig. 1c). However, the polyclonal antibody 8-1^15^, which recognizes the ectodomain of APLP2 stained soluble APLP2 fragments (designated as sAPLP2α/β in the conditioned medium of cells overexpressing APLP2), but fails to distinguish between sAPLP2α and sAPLP2β (Fig. 1c,d). Similarly, the polyclonal antibody against the ectodomain of APLP1 (42464;^15^) recognizes both forms, sAPLP1α and sAPLP1β (referred to as sAPLP1α/β in Fig. 1e,f).

The generation of secreted sAPLP2α/β and sAPLP1α/β is reduced in the presence of either α- or β-secretase inhibitors, with the α-secretase inhibitor having seemingly a stronger impact on levels of sAPLP2α/β and sAPLP1α/β (Fig. 1c–f). Surprisingly, in the medium of APLP1-overexpressing cells, a larger fragment appeared that is detected by an antibody directed against the Aβ-like region of APLP1 (A1β1–28; Fig. 1e,g). This fragment is exclusively associated with APLP1 in the absence and presence of α/β inhibitors. A similar fragment derived from APP or APLP2 could not be detected in media of overexpressing cells by the respective APP or APLP2 antibodies 4G8 (αAβ17–24) or 907899 (αA2β). Under the condition of γ-secretase inhibition the formation of the fragment is abolished which strongly indicates that γ-secretase activity is involved in its generation (Fig. 1e,f). APLP1-overexpressing SH-SY5Y cells also released the sAPLP1γ fragment and secretase inhibitor experiments had similar effects on secretory forms as observed with HEK293T cells (Fig. 1g,h vs. 1e,f). These results suggest that γ-secretase cleaves APLP1 without prior processing by α- or β-secretase and implies that, unlike other APP family members, APLP1 is an immediate substrate of γ-secretase. The novel fragment is hereinafter referred to as sAPLP1γ.

To investigate whether the observed effect on APLP1 γ-shedding was due to overexpression, we analyzed APLP1 processing in wild-type human neuroblastoma (SH-SY5Y) cells. To detect APLP1 in the conditioned medium of wild-type (i.e. non-transfected) SH-SY5Y cells, sAPLP1 was immunoprecipitated with the APLP1 ectodomain-specific polyclonal antibody αAPLP1ecto and subsequently detected using 42464 (for sAPLP1α/β), αA1β1-28 (for sAPLP1γ), or 907892 (for flAPLP1) (Fig. 1i). Western blot analyses of endogenous APLP1 from SH-SY5Y cells treated with or without γ-secretase inhibitor show that the secretion of sAPLP1α/β is independent from inhibitor treatment whereas sAPLP1γ is below the detection limit when γ-secretase is inhibited. Of note, full-length forms of APP and APLPs released into the supernatant could not be detected in the cell culture supernatants from SH-SY5Y or HEK293T cells.

### Modulation of α-, β-, and γ-secretases affects the generation of sAPLP1γ

**Figure 2:**
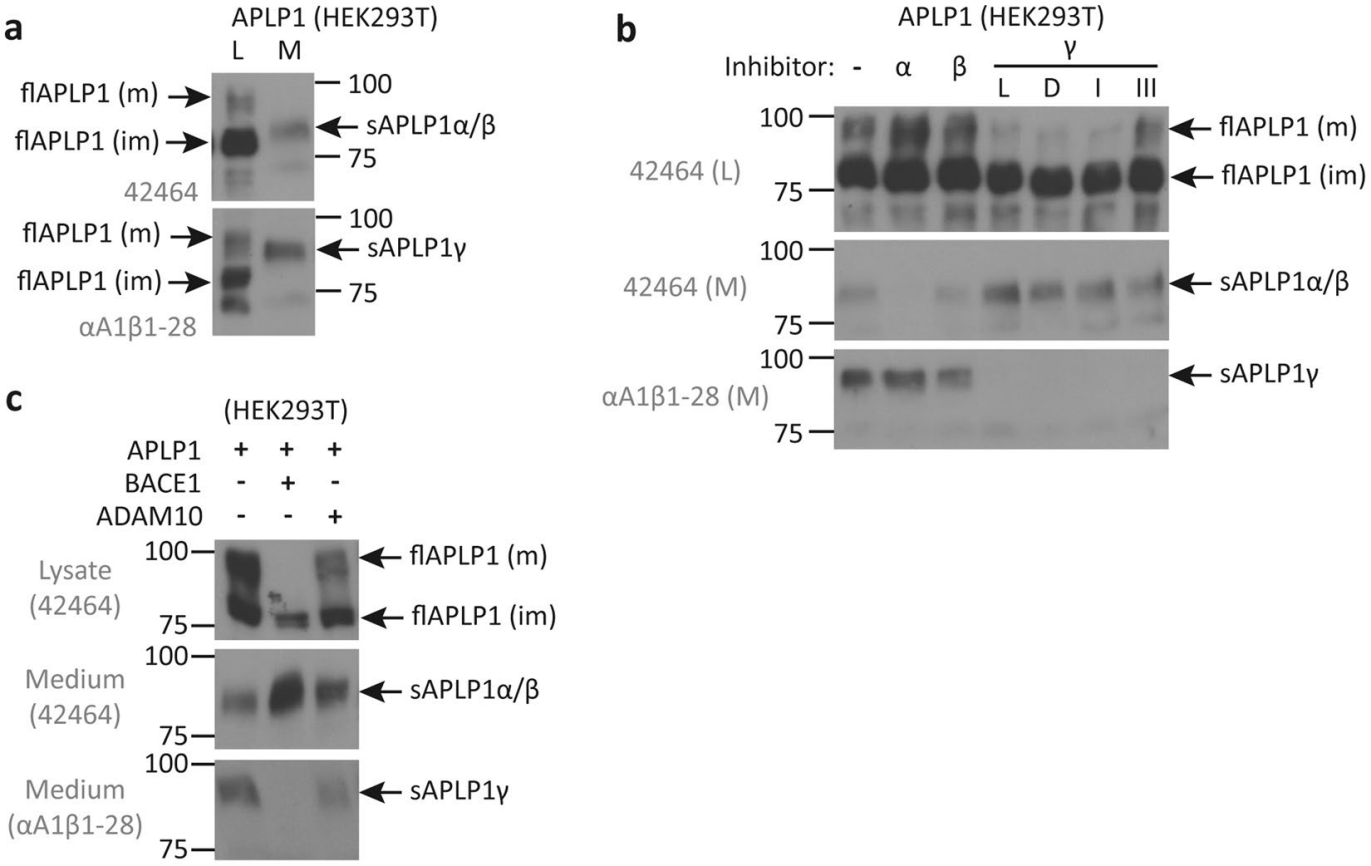
Western blot analysis of soluble and full-length APLP1 species. Conditioned medium (M) or lysate (L) of HEK293T cells overexpressing APLP1 was analyzed with the anti APLP1 antibodies 42464 and αA1β1–28 to display differences in size between sAPLP1γ, fl-APLP1 and sAPLP1α/β and between mature (m) and immature (im) full-length APLP1 (**a**). In HEK293T cells overexpressing APLP1 treated with α-, β- or different γ-secretase inhibitors (α – GM6001; β – β-secretase-inhibitor IV; γ/ L – L-685,458; D – DAPT; I – GSI I; III – GSI III), full-length and secreted forms were analyzed in L and M with the antibodies as indicated (**b**). HEK293T cells were co-transfected with APLP1 and BACE1 or ADAM10 and full-length and secreted forms analyzed in L and M with the indicated antibodies (**c**). Displayed are representative Western blots of two independent experiments. Full-length blots of both experiments are presented in Supplementary Figures 2 and 3.

To differentiate between soluble sAPLPγ and full-length APLP1 in the lysate and medium of APLP1 transfected HEK293T cells we performed Western blot analyses with the antibodies 42464 and αA1β1-28 that recognize both forms. The band corresponding to sAPLPγ migrates between the strongly glycosylated mature and the core-glycosylated immature full-length APLP1 and slightly higher than the shorter fragments sAPLP1α/β that do not bear the epitope sequence of αA1β1-28 (Fig. 2a; see Methods section).

To test, whether sAPLP1γ generation might depend on the molecular properties of the γ-secretase inhibitor used, we analyzed the expression and the processing of APLP1 in the presence of L-685,458 (a transition-state analog), DAPT (N-[N-(3,5-difluorophenacetyl)-L-alanyl]-S-phenylglycine t-butyl ester; dipeptidic type;^37^), and the peptidic inhibitors GSI I and III. All four γ-secretase inhibitors prevented sAPLP1γ release in a similar manner, whereas sAPLP1α/β levels were found enhanced in the medium compared to the control and α- and β-secretase inhibitor treatments (Fig. 2b). Additionally, γ-secretase inhibitor treatment decreased the levels of mature APLP1 (flAPLP1(m)) relative to immature APLP1 (flAPLP1(im)), possibly promoting the internalization and/or α/β-secretase-mediated processing of flAPLP1(m). Especially α-secretase inhibition led to a moderate increase of sAPLP1γ in the supernatant and to an increase of flAPLP1(m) levels in the cell lysate (Fig. 2b), which implies competition between the α-cleavage and γ-cleavage for membrane anchored APLP1 as the direct substrate.

Because there were differences between the α- and β-secretase inhibitors regarding sAPLP1γ generation, we also wanted to assess the effects of an increased ratio of enzyme to substrate ^_^ i.e., analyzing increased activities of ADAM10, the main α-secretase^38,39^, or BACE1 in APLP1-overexpressing cells. The results reveal that overexpression of BACE1 converts flAPLP1(m) into sAPLP1β with no residual amounts of sAPLP1γ detectable, while ADAM10 overexpression leads to significantly attenuated levels of flAPLP1(m) and detection of residual amounts of sAPLP1γ (Fig. 2c).

Taken together, these findings reveal a novel secreted fragment of APLP1, i.e. sAPLP1γ, released from APLP1- overexpressing cells (HEK293T and SH-SY5Y) and from wild-type SH-SY5Y cells after enrichment of sAPLP1γ by immunoprecipitation. Thus, sAPLP1γ is uniquely generated by γ-secretase from full-length APLP1, mainly in a competing reaction between the classical sheddases (BACE1, ADAM10) and γ-secretase, whereas corresponding fragments of APP or APLP2 were not detected.

### APLP1 is cleaved after Leu595 to release sAPLP1γ

**Figure 3:**
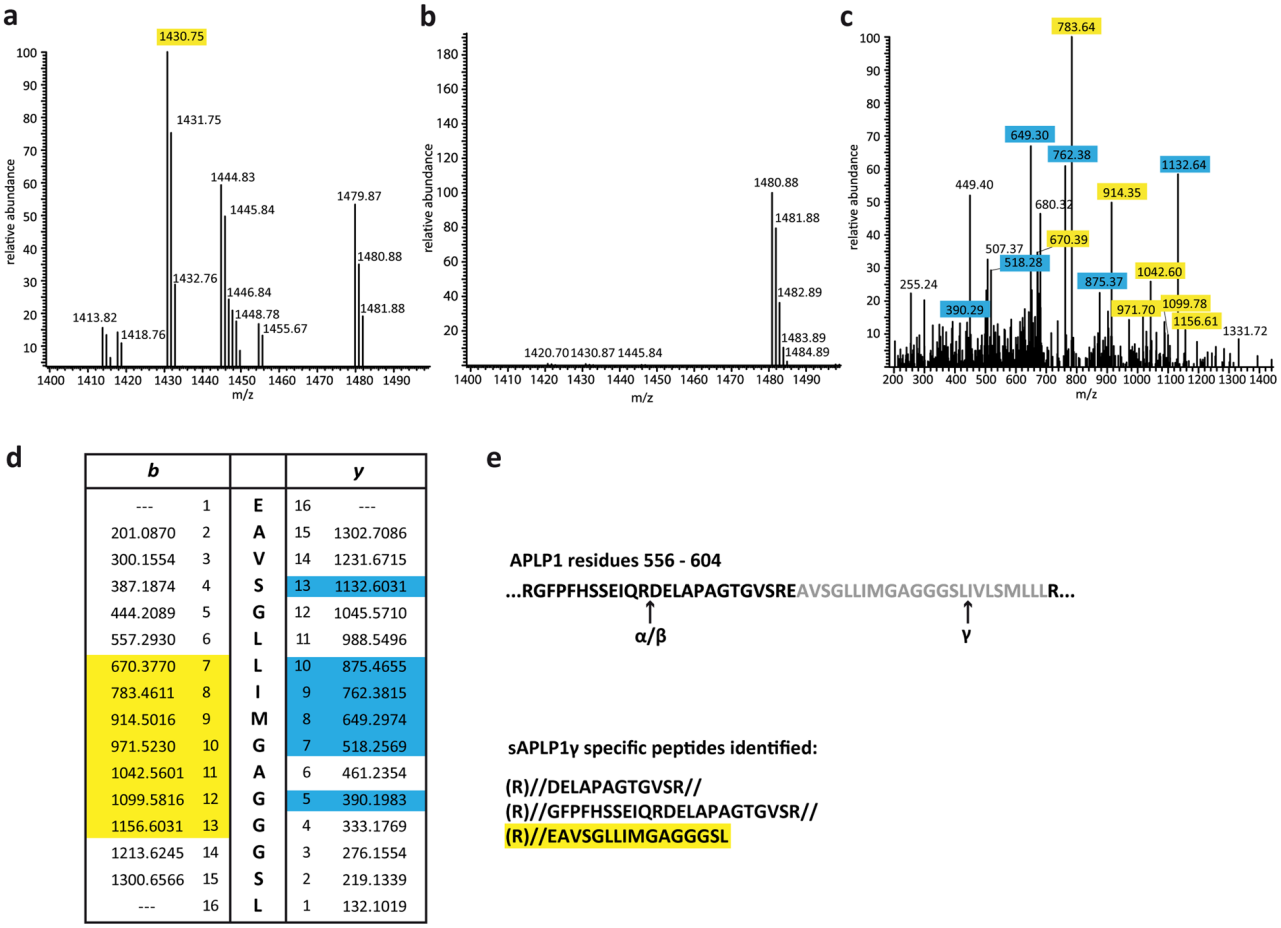
LC-MS analysis to identify the sAPLP1γ C-terminus. Tryptic peptide spectrum of sAPLP1γ (**a**) and sAPLP1α/β (**b**) at about 1400 Da. The peak at 1430.75 only appears in sAPLP1γ samples. MS/MS analysis of the sAPLP1γ peptide at m/z = 1430.75 Da (**c**) and comparison with expected b and y sequence fragments of the APLP1 peptide EAVSGLLIMGAGGGSL (**d**). The peptides highlighted in yellow (*b*) and blue (*y*) were detected. APLP1 sequence 556–604 with indicated α-, β- and γ-cleavage sites (TMS in grey) and list of tryptic and semi-tryptic peptides from that sequence found in sAPLP1γ samples (**e**).

Since sAPLP1γ migrated at a higher molecular weight compared to sAPLP1α/β (Fig. 2a), we expected the γ-secretase-mediated cleavage to occur within or close to the TMS. To determine the exact cleavage site by mass spectrometry, N-terminally His-tagged APLP1 was overexpressed in HEK293T cells and treated with α- and β-secretase inhibitors to specifically increase secretion of sAPLP1γ or with γ-secretase inhibitor to increase levels of sAPLP1α/β as a control, respectively. The soluble APLP1 species were enriched by Ni-NTA affinity chromatography and eluates were subjected to SDS-PAGE. The bands corresponding to sAPLP1γ and sAPLP1α/β were cut out from the gel and peptides generated by tryptic in-gel digestion were analyzed by liquid-chromatography mass spectrometry (LC-MS). To determine the C-terminal residue of sAPLP1γ (i.e. the cleavage site of the γ-secretase), all identified peptides were run against a database of non-specifically cleaved APLP1 peptides. This approach yielded a semi-tryptic peptide of 1430.75 Da that was only found in sAPLP1γ (Fig. 3a), but not in sAPLP1α/β control samples (Fig. 3b). By MS/MS analysis the sequence of this peptide could be determined as EAVSGLLIMGAGGGSL which corresponds to the C-terminal part of the A1β1-28 sequence (Fig. 3c,d). Thus, the γ-secretase cleavage site in full-length APLP1 is identical to the one in previously α/β-cleaved APLP1 C-terminal stubs^25^. Sequences extending beyond the known α- or β-secretase cleavage site were only identified in sAPLP1γ samples but not in sAPLP1α/β samples ending with amino acid 567 (Fig. 3e). This novel observation further confirms the unique cleavage of full-length APLP1 by the γ-secretase in addition to previously identified secretase cleavage sites in APLP1.

### The APLP1 transmembrane sequence is necessary and sufficient for γ-shedding and release of full-length APLP1

**Figure 4:**
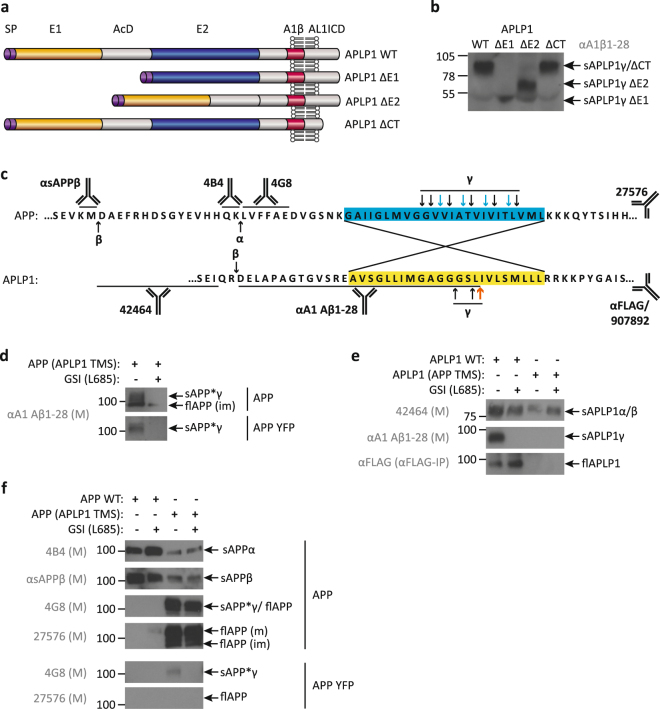
Soluble processing products of APLP1 deletion mutants and APLP1-APP chimeras. Schematic representation of the APLP1 domains and APLP1 deletion mutants analyzed: SP – signal peptide; E1 and E2 – two conserved regions of the ectodomain; AcD – acidic stretch linker region between E1 and E2; A1β – amyloid beta-like sequence of APLP1; AL1ICD – APLP1 intracellular domain; CT – C-terminus (**a**). Conditioned media of HEK293T cells overexpressing the APLP1 deletion mutants, detection of sAPLP1γ species with the polyclonal antibody αA1β1–28 (**b**). Amino-acid sequences of APP and APLP1 around the transmembrane sequence (TMS – yellow or blue box), indicated are the cleavage sites of α-, β- and γ-secretase and the recognition sites of the antibodies used (**c**). For the creation of the chimeric proteins the TMS of APLP1 was inserted into the APP sequence and *vice versa* to replace the respective TMS. Analysis of the conditioned media of HEK293T cells overexpressing WT or chimeric APP and APLP1. Soluble species and full-length forms in the conditioned media of HEK 293T cells overexpressing APP WT and APP (APLP1 TMS) with or without C-terminal YFP tag were detected in the presence or absence of the γ-secretase inhibitor L – L-685,458 (L685) with the APLP1-specific antibody αA1β1–28 (**d**), APLP1 WT and APLP1 (APP TMS) with the antibodies 42464 and αA1β1–28 as well as αFLAG antibody after IP against the C-terminal FLAG tag (**e**) and with the APP-specific antibodies 4B4, αsAPPβ, 4G8 and 27576 (**f**). Displayed are representative Western blots of at least two independent experiments. Full-length blots are presented in Supplementary Figures 4 and 5.

Previously, we found that APLP1 ectodomain shedding of zinc-induced APLP1 oligomers was rescued by deletion of the E2 domain^16^. Thus, we then tested whether sAPLP1γ generation is changed by APLP1 mutants lacking the E1 and acidic domain AcD (ΔE1), the E2 domain (ΔE2) or the cytosolic domain (ΔCT) (Fig. 4a).

All mutant derivatives of APLP1 were secreted into the supernatant and no obvious qualitative alterations were detected in the generation of respective sAPLP1γ forms between the mutants (Fig. 4b). As such, we consequently assessed whether the TMS of APLP1 plays a decisive role (Fig. 4c). To address this question, we created chimeric proteins (i.e. APLP1 with the TMS of APP, termed APLP1 (APP TMS)) as a potential negative control. *Vice versa*, the suitability of APP with the APLP1 TMS, termed APP (APLP1 TMS), as a direct substrate of γ-secretase was tested under the assumption that the APLP1 TMS interaction with the enzyme is not influenced by ectodomain or cytosolic residues of APP.

We found that the chimera APP (APLP1 TMS) is directly cleaved by the γ-secretase and the respective mature soluble product sAPP*γ is detected in the supernatant by the polyclonal APLP1 antibody αA1β28 in the absence but not in the presence of the γ-secretase inhibitor (Fig. 4d). This demonstrates that the APLP1 TMS confers the specificity of the direct γ-secretase cleavage. Furthermore, the differentiation between the immature full-length chimera (flAPP(im)) which is generated independently from γ-secretase inhibitor treatment, demonstrates the existence of two separate pathways (Fig. 4d). Similarly, the YFP-tagged version of sAPP*γ derived from APP (APLP1 TMS) is only detected in the absence of the γ-secretase inhibitor and further indicates the specificity of sAPP*γ production from APP (APLP1-TMS) (Fig. 4d).

Using antibodies directed against the APLP1 ectodomain to analyze processing and release of the chimera APLP1 (APP TMS) processing and its release, we found that the APP TMS prohibits a direct cleavage by the γ-secretase (Fig. 4e). However, the chimera still secretes sAPLP1α/β, implying that the APP TMS had no impact on the cleavage by α/β-secretases (Fig. 4e). The attenuated levels of sAPPα/β derived from APP (APLP1 TMS) α/β-cleavage are due to an impaired secretion process as indicated by the high levels of the respective full-length forms (flAPP (m) and flAPP (im)) (Fig. 4f). Full-length wild-type APLP1 (flAPLP1) in cell culture supernatant was enriched by immunoprecipitation and detected with the C-terminal short FLAG-tag antibody, independent from γ-secretase inhibition (Fig. 4e). The YFP tag of APP (APLP1 TMS) permitted the detection of the derived sAPP*γ by the 4G8 antibody in the supernatant only in the absence of the γ-secretase inhibitor. The long C-terminal YFP tag apparently prohibited the release of the YFP-tagged full-length forms as indicated by the failed detection of flAPP with the APP antibody 4G8 and 27576 (Fig. 4f). Of note, the untagged APP (APLP1 TMS) chimera co-migrates with sAPP*γ and is released as mature and immature flAPP detected by the C-terminal antibody against APP (27576) (Fig. 4f).

The APLP1 TMS is critical for the generation and secretion of sAPLP1γ into the supernatant. While the phenomenon of full-length APLP1 release was previously described by others^24,33^, we show here that the release of full-length forms is solely dependent on the TMS of APLP1, enhanced by surrounding APP sequences (compare flAPLP1 in Fig. 4e with flAPP in Fig. 4f), and independent of γ-secretase activity. Overall, the APLP1 TMS is sufficient to convert the type-I transmembrane protein APP into a direct γ-secretase substrate.

## Discussion

APP and the APP-like proteins (APLP1, APLP2) are multidomain transmembrane proteins that exhibit adhesive and signaling activities within the nervous system. The Aβ sequence is unique to APP but, co-expression of APP in individual combinations with APLPs modulates Aβ production^15,16^. We have previously shown that the APLP1 protein mainly localizes to the cell surface whereas the two other protein family members (APP, APLP2) are mostly found inside the cell^15^.

All three proteins exist as membrane-bound and secreted forms and possess homo- and heterotypic contact sites in their ectodomains^4,15,40^. While we confirm here that BACE1 is a major sheddase for APLP1, the loss of BACE1 processing of APP, APLP1 and APLP2 had no detrimental effect on the *de novo* production of ICDs^24^. However, BACE1-initiated APLP1 processing generated sAPLP1β, APL1β, and AL1ICD^27,32^. Together, these findings suggest the existence of another, yet unrecognized, major pathway for APLP1 processing and AL1ICD generation. We show here that, in the novel pathway, sAPLP1γ is directly generated by γ-secretase from full-length APLP1 and released into the cell culture supernatant. Thus, our new findings challenge our current understanding of APP and APLP1 processing and the role of APLP1-derived fragments in AD^41^.

We found sAPLP1γ in wild-type SH-SY5Y cells (a common *in vitro* model for endogenous APLP1 expression and neuronal function) and in APLP1-overexpressing HEK293T and SH-SY5Y cells. Without finding a similar or equivalent fragment for APP or APLPL2, we are the first to describe this unique feature of APLP1 to our knowledge. Surprisingly, this novel mechanism of endoproteolytic cleavage exerted by γ-secretase is consistent with an ϵ-cleavage event based on the endoproteolytic nature of the reaction, which was originally designated for the liberation of the ICD from CTFs of APP by the γ-secretase^42^. The ϵ-cleavage occurs between amino acids 48-49 or 49–50 resulting in two AICDs (AICD49 and AICD50, respectively). An alternative additional cleavage site is created when Gly33 is mutated to Gln which produces a shift and generates AICD52^43,44^. Historically, the ϵ-cleavage had been assumed to occur in the middle of the APP TMD to generate Aβ42. However, for APP – and likewise for APLP1 and APLP2 – the shedding of the ectodomain by β- or α-secretase is mandatory before the respective CTF becomes a substrate for γ-secretase^45^. While APLP1 can undergo processing by β- and γ-secretase to form short Aβ-like peptides^24,25,46,47^ such as APL1β25, APL1β27, and APL1β28, we also now demonstrate direct γ-secretase processing of APLP1.

Using an N-terminally His-tagged construct and affinity purification, we identified the C-terminal sequence of sAPLP1γ by mass spectrometry. The peptide EAVSGLLIMGAGGGSL found in sAPLP1γ, but not in sAPLP1α/β, is not a regular tryptic peptide. It shares the C-terminal amino-acid sequence with A1β1–28, i.e. the γ-secretase product of APLP1-CTF cleavage. Thus, full-length APLP1 is cleaved after TMS residue Leu595 to release sAPLP1γ as determined by MS/MS analysis, which requires an intramembrane-cleaving protease. The generation of sAPLP1γ can be blocked by various γ-secretase inhibitors. In addition to the pharmacological approach and controls provided by nature, i.e. APLP2 and APP, we used genetic approaches and showed that a potential similar fragment derived from APLP1 bearing the APP-TMS could not be detected. Moreover, when we tested the APP chimera (APP bearing the APLP1 TMS) construct, the resulting protein was directly cleaved by γ-secretase. While these arguments indicate that the cleavage is γ-secretase specific, gene silencing experiments and/or a transfection of γ-secretase could provide information what proteolytic fragments of APLP1 are important for what biological function and how sAPLP1γ generation is controlled by varying levels of γ-secretase activity.

As demonstrated in the present study, this finding represents an alternative mechanism for AL1ICD generation in which the α/β-cleavage steps are by-passed. This process excludes the generation of APLP1-derived Aβ-like peptides from membrane-bound stubs and predicts the existence of an AL1ICD that starts with amino acid Ile 596 (Fig. 3e), five residues N-terminal to the Leu 601 site that was predicted based on similarity with the APP sequence^31^. The longer sequence of AL1ICD, compared to AICD forms^43^, might also explain why AL1ICD does not translocate to the nucleus despite binding to Fe65^31^, and does not have a direct nuclear signaling function like the ICDs of APP and APLP2^31^. Also, our newly proposed mechanism answers the long-standing question as to why AL1ICD levels were found to be slightly higher in extracts of BACE1 KO brains^24^ - i.e. due to the direct and efficient γ-secretase cleavage of APLP1. AL1ICD levels might be slightly lower in AD brains, since BACE1 protein levels are significantly elevated in AD, the levels and enzymatic activity of BACE1 are increased in prodromal AD/AD brain tissue^48,49^ and CSF^50–52^. Given these results, varying APLP1/APP:BACE1 ratios could influence the AL1ICD levels as well as interaction partners of the γ-secretase complex^53^. Moreover, studies have provided strong evidence that the zinc metalloprotease meprin β can be considered an alternative β-secretase^54^. Meprin β could also influence AL1ICD levels since mutations near the start of the Aβ sequence were also found to alter the efficiency of meprin β cleavage of APP. The protective alanine to threonine mutation at the p2 position of the Aβ sequence identified as the first protective mutation against AD^55^ inhibits both BACE1 cleavage as well as meprin β cleavage^56^. Other factors that could determine the pathway of AL1ICD production are clustering mechanisms of APP and APLPs that impair APLP1 processing by secretases leading to reduced levels of soluble APPs and APLPs^16^. Overall, the endoproteolytic cleavage by γ-secretase, and the subsequent release of sAPLP1γ, represents a new pathway in which BACE1 and γ-secretase compete for the processing of APLP1.

**Figure 5:**
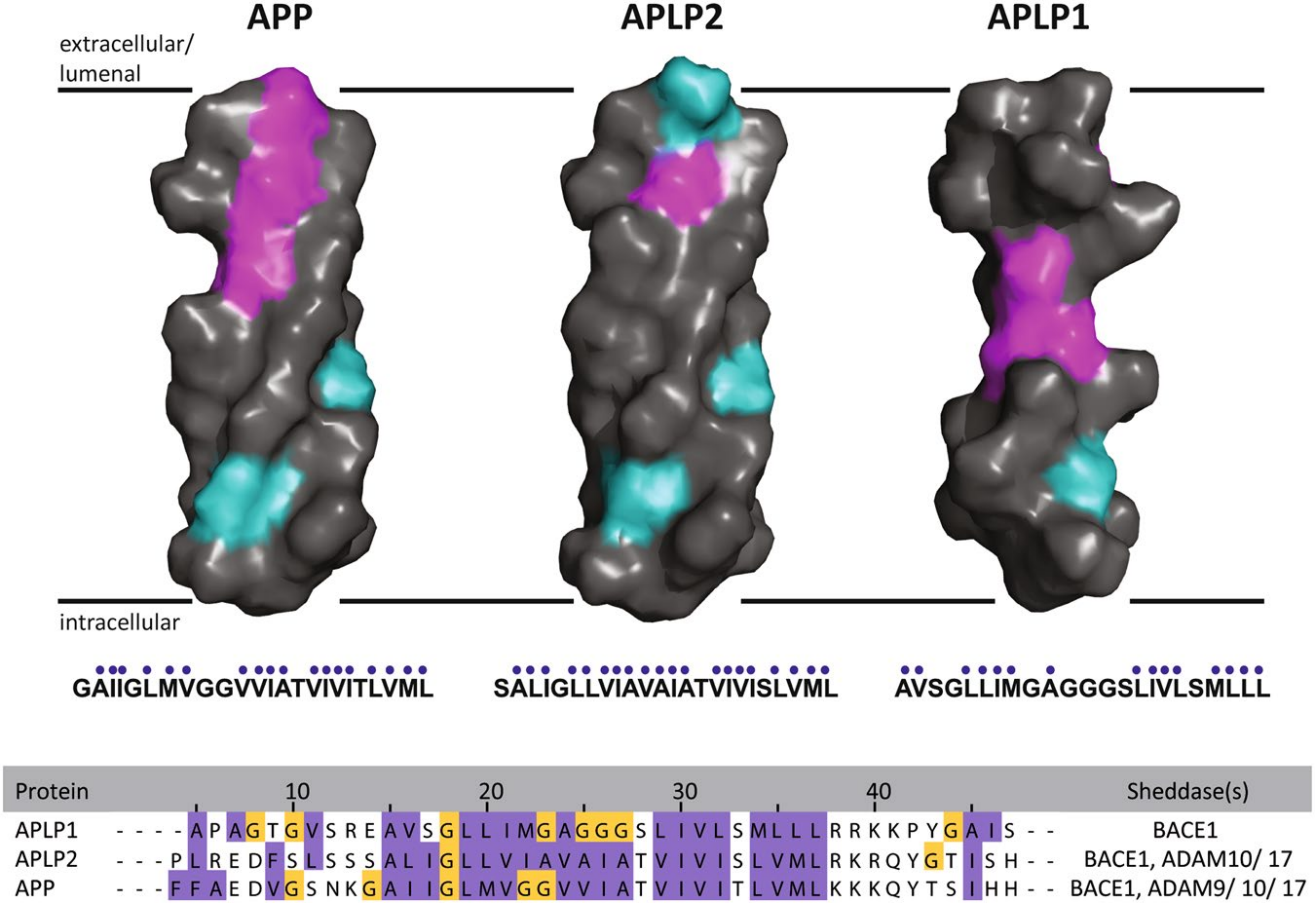
Molecular model of the APLP1 TMS within the plasma membrane. The APLP1 TMS has a unique sequence and structural flexibility within the membrane**:** side-view of 3D surface representations of the putative transmembrane sequences of APP family members, assuming ideal α-helices. Hydrophobic amino acids are colored in gray, polar uncharged amino acids in cyan, and glycine in magenta. The area around the respective TMS of APP and APLPs (hydrophobic amino acids are highlighted in purple, glycines in yellow) sequences were aligned using Clustal Ω and JalView. Sheddases which are known to cleave the respective substrate are listed (ADAMs; BACE1).

The only report of a transmembranous substrate that is directly cleaved by γ-secretase is the short B-cell maturation antigen mBCMA with only 54 amino-acid residues^57^. Thus, based upon the known homo- and hetero-interaction sites in the ectodomain of APLP1^15,16,21^, we tested the possibility that specific domains, or in general, a size-reduced ectodomain could qualitatively cause changes in sAPLP1γ generation. Though the exact cleavage site in mBCMA was not mapped^57^, the small size of the protein provided the only explanation for how mBCMA can be an immediate γ-secretase substrate. As previously postulated by Hemming and co-workers^58^, their definition of γ-secretase substrates (i.e. a short ectodomain which is directly cleaved, and ectodomain shedding to instruct subsequent γ-secretase processing) did not apply in the case of APLP1. In our present study, we found that neither the shortening of the large ectodomain nor the deletion of the small cytoplasmic domain of APLP1 affected the direct cleavage of APLP1. This result left the TMS of APLP1 as the only remaining candidate region possessing the necessary sequence. Consequently, we tested the TMS of APLP1 and used chimeric constructs, APLP1 with the TMS of APP and APP with the APLP1 TMS. We chose APP since it was not found to serve as a direct substrate of γ-secretase and has a TMS of similar length (24 residues vs. 23 in APLP1). Both TMSs are rich in glycine residues and both contain GxxxG motifs, termed as glycine zippers^59^, which are not found in APLP2. The GxxxG motif is known to mediate helix–helix interactions in the APP TMS in a consecutive arrangement^60–62^. In APLP1, a variation of the GxxxG motif is present (G/AxxxG/S) but a possible involvement in helical interactions has not been reported. Our data now show that the APLP1 TMS successfully converts APP into a γ-secretase substrate, while *vice versa* the TMS of APP in APLP1 prohibits direct cleavage by γ-secretase while α/β-secretase activities are not grossly altered. The 3D molecular views of the APP and APLPs TMSs (Fig. 5) rendered using PyMOL^63^ illustrate a Gly-rich region of the APLP1 TMS in the center of the helix. In general, amino-acid sequences rich in glycines tend to form coils^64^, allow higher degrees of structural flexibility and create swivels and hinges as the model in Fig. 5 predicts for the APLP1 TMS. The APLP1 stretch of 6 amino acids containing 4 glycine residues (GAGGGS) may form a GxxxG motif partially overlapping in itself by extending the actual GxxxG motif either to the N- and/ or the C-terminus including the Ala and Ser residues^59^. Moreover, the APLP1 TMS possesses a high ratio of glycine residues to hydrophobic residues, 0.33 compared to 0.05 in APLP2 and 0.22 in APP. The APLP1 Gly-cluster – absent in APP and APLP2 – might weaken the insertion into the membrane supported by a ‘hydrophobic gap‘, filled with helix-destabilizing glycine residues that enhance the structural variability of the TMS (Fig. 5). Similarly, the Gly-cluster might also enable the release of full-length APLP1 from the membrane, another unexplained observation in the literature^24^. The release occurred independently from γ-secretase activity and was prohibited by an extended tag of an engineered APLP1 construct and facilitated by surrounding APP residues of the APLP1-TMS chimera. As for APLP1, the TM regions of plexin-A1, A2, A3 and A4^65^ are characterized by poly-glycine motifs in the membrane interior that distort helix conformation and association: GGGGG in A1, GGG in A3, GG in A2 and A4. Molecular dynamics simulations of plexin-A1 showed a partial unfolding at this position, allowing a more extended structure (plexin-A family members have 22 TM residues, which is similar to APLP1 with 23 and APP with 24 residues). Regardless, recognition of the substrate TMS *per se* and its processing by γ-secretase is dictated by the TMS sequence upstream from the ε-cleavage site^66^ which in the case of APLP1 is the GAGGGS sequence (Fig. 5).

Taken together, our present study provides critical new insight about the three APP family members: direct cleavage of APLP1 is a novel feature of the γ-secretase that by-passes prior ectodomain shedding. The finding that the APLP1 TMS, rather than the large ectodomain, is the determining factor for sAPLP1γ production requires a re-thinking of γ-secretase activity modulation including a re-definition of the substrate features of the γ-secretase. Our data adds another set of unsolved questions, for example how does γ-secretase recognize substrates in general and transfer full-length APLP1 to the active site? As is to be expected from our results with γ-secretase inhibitors, how would AD-linked PS1 mutations and γ-secretase modulaters (GSMs) targeting the γ-secretase active site modulate the cleavage of full-length APLP1 and what would be the consequences for AD development and such treatment approaches?

Finally, the importance of the APLP1 TMS sequence helps explain the previously observed but unaccounted for release of full-length APLP1 from cells that occurs independently from γ-secretase activity.

## Methods

### Expression plasmids

APP, APLP2 as well as APLP1 WT, APLP1 ΔE1-AcD, ΔE2 and ΔCT cDNA was ligated into custom made vectors pcDNA3-FLAG, pcDNA3-CFP and pcDNA3-YFP as previously described^16,61^. The insertion of the N-terminal His_6_-tag in the APLP1 sequence after the signal peptide (aa1–38) was carried out by PCR based site-directed mutagenesis using partially overlapping primers (Zheng *et al*., 2004). APLP1-APP chimeras were created by an inverse PCR technique (Hemsley *et al*., 1989). The transmembrane sequence of APP695 (aa635–658) was inserted into APLP1 instead of the APLP1 TMS (aa581–603) and vice versa. Primers were designed to cover one half of the inserted TMS and the corresponding adjoining sequence in the vector. The sequences of all PCR or mutagenesis fragments were verified by DNA sequencing (GATC).

### Cell culture

HEK293T cells and SH-SY5Y cells were grown in an incubator at 37 °C and 5% CO_2_. The HEK293T growth medium was DMEM (PAA) supplemented with 10% FCS (PAA), 2 mM glutamine and 1 mM sodium pyruvate. SH-SY5Y cells were grown in DMEM/Ham’s F12 (Biochrom) supplemented with 10% FCS (PAA), 2 mM glutamine, 1 mM sodium pyruvate and 1× non-essential amino acids (PAA). Cells were transfected with the indicated plasmids using polyethylenimine or Transfectin transfection reagent (BioRad). The cell culture medium was exchanged 18 hours after transfection. The newly added medium contained the indicated inhibitors and was conditioned for 6 hours. Activity of following secretases was inhibited by specific inhibitors from Calbiochem: α-secretase (GM6001; 50 µM), β-secretase (β-secretase inhibitor IV; 20 µM) and γ-secretase (L-685,458; DAPT; γ-secretase inhibitor I and III; each at 10 µM).

### Immunoprecipitation and immunoblots

C-terminally FLAG tagged proteins in the conditioned medium were immunoprecipitated by adding 2 µl M2-FLAG antibody (Sigma) and 30 µl protein G sepharose (GE Healthcare) and incubated overnight in a rotating shaker at 4 °C. Soluble APLP1 species in the conditioned medium of untransfected SH-SY5Y cells were specifically enriched by overnight incubation with 5 µl of the polyclonal antibody αAPLP1ecto (R&D Systems) and 30 µl protein G sepharose at 4 °C. After centrifugation sepharose pellets were washed thrice with buffers with decreasing salt concentration and finally resuspended in gel loading buffer. Samples were subjected to SDS-PAGE and transferred to nitrocellulose membranes (Machery & Nagel). Immunodetection was performed using antibodies specific for the respective protein, followed by probing with horseradish peroxidase-coupled anti-mouse (1:10,000; Promega), anti-rabbit (1:10,000; Promega), anti-rat (1:10,000; Promega), or anti-goat IgG antibodies (1:1,000; R&D Systems), respectively. Chemiluminescence was detected using ECL reagent. Band intensities were quantified by densitometry with ImageJ.*Antibodies used in the study together with their source and the epitope recognized*. ***Antibody name***, *Antibody target*, *Source*, *Epitope recognized*, *Obtained from:* **αsAPPβ**, Human sAPPβ, Rabbit polyclonal, C-terminus of human sAPPβ, IBL, Japan. **4B4**, Human sAPPα, Rat polyclonal, C-terminus of human sAPPα, Kuhn *et al*. 2010; gift from Dr. Lichtenthaler. **W0–2**, Human Aβ, Mouse monoclonal, Human Aβ residues 4–10, Multhaup lab, EMD Milipore. **4G8**, Human Aβ, Mouse monoclonal, Human Aβ residues 17–24, Covance, USA. **27576**, Human APP, Rabbit polyclonal, C-terminus of human APP (residues 648–695), Multhaup lab. **42464**, Human APLP1, Rabbit polyclonal, Ectodomain of human APLP1 (residues 499–557), Multhaup lab. **αAPLP1ecto**, Human APLP1, Goat polyclonal, Ectodomain of APLP1 (residues 34–580), R&D Systems, USA, AF3129. **αAPL1β1–28**, Human APL1β1–28, Rabbit polyclonal, Human APL1β1–28 (residues 568–595), Bachem, USA. **907892**, Human APLP1, Rabbit polyclonal, C-terminus of human APLP1, Multhaup lab. **8–1**, Human APLP2, Rabbit polyclonal, Ectodomain of human APLP2, Multhaup lab. **907899**, Human APL2β, Rabbit polyclonal, Human APL2β, Multhaup lab. **907779**, Human APLP2, Rabbit polyclonal, C-terminus of human APLP2, Multhaup lab.

### Purification of soluble APLP1 species

HEK293T cells were transfected with pcDNA3-N-His-APLP1 and treated with either γ-secretase inhibitor L-685,458 or α-secretase inhibitor GM6001 and β-secretase inhibitor IV. The over 6 hours conditioned medium was applied to Ni-NTA columns (Machery & Nagel). Columns were washed with binding buffer containing 50 mM imidazole and bound proteins were eluted with 250 mM imidazole. The APLP1-containing elution fractions identified by dot blot were combined and concentrated with Amicon filter units. The proteins in the concentrated eluates were separated by SDS-PAGE and stained with Coomassie Brilliant Blue. Bands representing sAPLP1γ or sAPLP1α/β were isolated for LC-MS analysis.

### Trypsin digestion and liquid chromatography-electrospray ionization-tandem mass spectrometry (LC-ESI-MS/MS)

Proteins were separated by SDS-PAGE under reducing conditions, individual protein bands were excised and peptides were obtained by trypsin in-gel digestion as described previously (modified from^67^). The resulting peptide mixtures were separated by reverse-phase chromatography using a Dionex Ultimate 3000 nanoLC system (Thermo Fisher Scientific, Bremen, Germany) on in-house manufactured 25 cm fritless silica microcolumns with an inner diameter of 100 μm packed with ReproSil-Pur C18-AQ 3 μm resin (Dr. Maisch GmbH, Entringen, Germany) on a 5–60% acetonitrile gradient (90 min) with 0.1% formic acid at a flow rate of 350 nl/min. Eluting peptides were ionized on-line by electrospray ionization and transferred into an LTQ Orbitrap Velos mass spectrometer (Thermo Fisher Scientific, Bremen, Germany) operated in the positive mode to measure full-scan MS spectra (from m/z 300–1700) in the Orbitrap analyzer at resolution R = 60,000 followed by isolation and fragmentation of the twenty most intense ions in the LTQ part by collision-induced dissociation.

MaxQuant software (version 1.3.0.5) was used to process the raw MS files and the search engine Andromeda^68^ to compare the extracted peak lists against forward and backward protein sequences of the Uniprot Human reference proteome Database and 248 frequently observed laboratory contaminants. Initial maximum precursor and fragment mass deviations were set to 7 ppm and 0.5 Da, respectively. Methionine oxidation/acetylation of peptide N-termini and cysteine carbamidomethylation were set as variable and fixed modification, respectively for the search. The target-decoy-based false discovery rate (FDR) for peptide and protein identification was set to 1% for peptides and proteins and the minimum peptide length was set to 7 amino acids. No enzyme specificity was specified, so that all possible peptide fragments were included in the search.

### Molecular Modelling

3D representations of α-helical transmembrane sequences were generated using builder in PyMOL 1.7.4.

### Statistical analysis

Data are expressed as means ± Standard Error. All statistical analyses were performed by one-sample t-test vs. untreated control (100%) with Bonferroni correction. Western blots shown are representative of at least two independent experiments.

### Data availability

The data that support the findings of this study are available from the corresponding authors on reasonable request.

## Electronic supplementary material

Supplementary Information
