# Supplementary material for: APLP1 is endoproteolytically cleaved by γ-secretase without previous ectodomain shedding
Source: Sci Rep. 2018 Jan 30;8:1916. doi: 10.1038/s41598-018-19530-8 (PMC5789831; doi:10.1038/s41598-018-19530-8)
Supplement: Supplementary file 1 — Supplementary Information [file 41598_2018_19530_MOESM1_ESM.pdf]

Supplemental Information

APLP1 is endoproteolytically cleaved by γ-secretase without previous ectodomain shedding

Linda Schauenburg[1,§], Filip Liebsch[2], Murat Eravci[1], Magnus C. Mayer[1,#], Christoph Weise[1], Gerhard Multhaup[1,2†]

[1] Institut für Chemie und Biochemie, Freie Universität Berlin, Thielallee 63, 14195 Berlin, Germany

[2] Department of Pharmacology & Therapeutics and Integrated Program in Neuroscience, McGill University, 3655 Promenade Sir William Osler, Montreal, QC, Canada, H3G 1Y6

[§] present address: Sphingotec Therapeutics GmbH, Neuendorfstr. 15a, 16761 Hennigsdorf, Germany

[#] present address: Miltenyi Biotec GmbH, Robert-Koch-Strasse 1, 17166 Teterow, Germany

[†]**Corresponding Author:** Gerhard Multhaup, Tel.: (514) 398-3621, Fax: (514) 398-2045, E-mail: gerhard.multhaup@mcgill.ca

The following pages include:

Supplementary Figure legends

Supplemental Figure 1

Supplemental Figure 2

Supplemental Figure 3

Supplemental Figure 4

Supplemental Figure 5

Supplementary Figure Legends

Supplementary Figure 1: Number of actual repeats and representative full-length blots of cropped blots shown in Figure 1. (a) Blots with antibodies against APP (corresponding to Figures 1a-b), (b) Blots with antibodies against APLP2 (corresponding to Figures 1c-d), (c) Blots with antibodies against APLP1 (corresponding to Figures 1e-f), (d) Blots with antibodies against overexpressed APLP1 in SH-SY5Y cells (corresponding to Figures 1g-h), (e) Blots with antibodies against endogenous APLP1 (corresponding to figure 1i). Transfected and untransfected cells were treated with α- (GM6001, Calbiochem), β- (β-secretase inhibitor IV, Calbiochem) or γ-secretase inhibitor (L-685, Calbiochem). Conditioned media were analyzed using specific antibodies for the soluble ectodomains (APLP1 – 42464; APLP2 – 8-1; APP – W0-2 or αsAPPβ), the Aβ-region (APLP1 – αA1β1-28; APLP2 – 907899; APP – 4G8) or the C-terminus (APLP1 – αFLAG/ 907892; APLP2 – αFLAG; APP – 27576). M – conditioned medium, L – lysate, / (M) – conditioned medium of untransfected cells.

Supplementary Figure 2: Full-length blots of cropped blots shown in Figures 2 a and b. (a) Blots with antibodies against APLP1 and soluble APLP1 species in lysate (L) and medium (M) of APLP1 overexpressing HEK293T cells (corresponding to Figure 2a). (b) Blots with antibodies against APLP1 treated with different GSIs (corresponding to Figure 2b), (c) Blots with antibodies against APLP1 treated with different GSIs, repeated experiment (corresponding to Figure 2b). α – GM6001; β – β-secretase-inhibitor IV; γ – γ-secretase inhibitor, L – L685; D – DAPT; I – GSI I; III – GSI III; 42464 - antibody against the soluble APLP1 ectodomain, αA1β1-28 – antibody against the APLP1 Aβ-region; M – conditioned medium, L – lysate.

Supplementary Figure 3: Full-length blots of cropped blots shown in Figures 2c. (a) Blots with antibodies against APLP1 cotransfected with BACE1 or ADAM10 (at the right is the antiA1β1-28 blot at a shorter exposure time (“short”)). (b) Blots with antibodies against APLP1 cotransfected with BACE1 or ADAM10, repeated experiment; 42464 - antibody against the soluble APLP1 ectodomain, αA1β1-28 – antibody against the APLP1 Aβ-region; M – conditioned medium, L – lysate.

Supplementary Figure 4: Full-length blots of cropped blot of APLP1 deletion mutants shown in Figure 4b, at the right is the antiA1β1-28 blot at a shorter exposure time (“short”).

Supplementary Figure 5: Full-length blots of cropped blots of APLP1-APP chimeras shown in Figures 4d-f. (a) αA1β1-28 blots detecting γ-cleaved soluble domain of APP (APLP1 TMS) chimera without or with YFP-tag on the full-length protein (corresponding to Figure 4d), (b) Blots detecting soluble APLP1 species of APLP1 WT and APLP1 (APP TMS) chimera (corresponding to Figure 4e), (c) Blots detecting soluble APP species of APP WT and APP (APLP1 TMS), full-length proteins without YFP tag (corresponding to Figure 4f, upper blots “APP”), (D) Blots detecting soluble APP species of APP WT and APP (APLP1 TMS), full-length proteins with YFP tag (corresponding to Figure 4f, lower blots “APP YFP”). 42464 - antibody against the soluble APLP1 ectodomain, αA1β1-28 – antibody against the APLP1 Aβ-region; 4B4 - antibody against the sAPPα, 4G8 – antibody against the APP Aβ-region, 27675 – antibody against the APP C-terminus; M – conditioned medium, L – lysate, m – mature, im – immature.

Suppl. Fig. 1

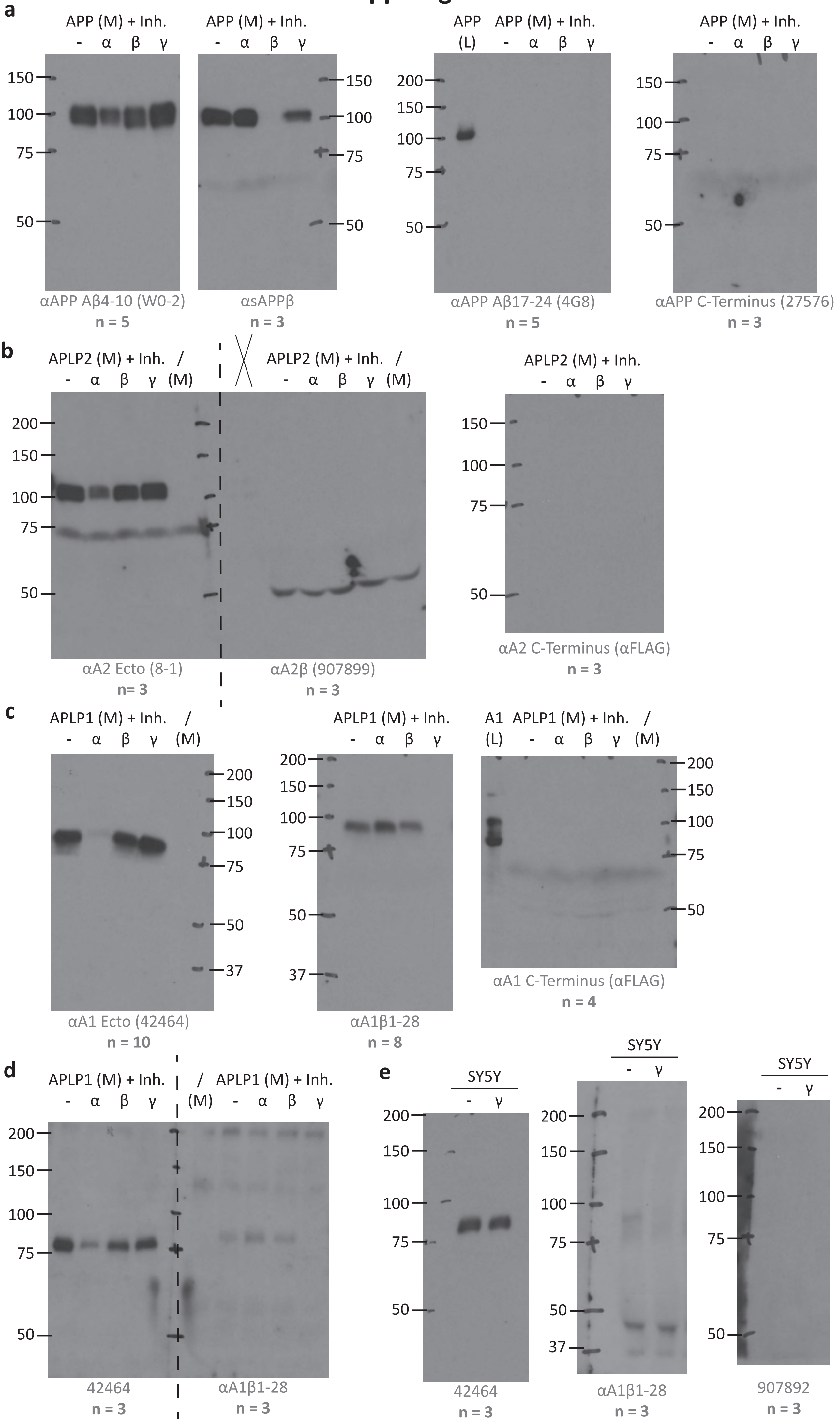
a
APP (M) + Inh.
- α β γ
150
100
75
50
αAPP Aβ4-10 (W0-2)
n = 5
APP (M) + Inh.
- α β γ
150
100
75
50
αsAPPβ
n = 3
APP (L)
APP (M) + Inh.
- α β γ
200
150
100
75
50
αAPP Aβ17-24 (4G8)
n = 5
APP (M) + Inh.
- α β γ
150
100
75
50
αAPP C-Terminus (27576)
n = 3
b
APLP2 (M) + Inh. /
- α β γ (M)
APLP2 (M) + Inh. /
- α β γ (M)
200
150
100
75
50
αA2 Ecto (8-1)
n= 3
αA2β (907899)
n = 3
APLP2 (M) + Inh.
- α β γ
150
100
75
50
αA2 C-Terminus (αFLAG)
n = 3
c
APLP1 (M) + Inh. /
- α β γ (M)
200
150
100
75
50
37
αA1 Ecto (42464)
n = 10
APLP1 (M) + Inh.
- α β γ
200
150
100
75
50
37
αA1β1-28
n = 8
A1 (L)
APLP1 (M) + Inh. /
- α β γ (M)
200
150
100
75
50
αA1 C-Terminus (αFLAG)
n = 4
d
APLP1 (M) + Inh.
- α β γ
/ (M)
APLP1 (M) + Inh.
- α β γ
200
150
100
75
50
42464
n = 3
αA1β1-28
n = 3
e
SY5Y
- γ
200
150
100
75
50
42464
n = 3
SY5Y
- γ
200
150
100
75
50
37
αA1β1-28
n = 3
SY5Y
- γ
200
150
100
75
50
907892
n = 3

Suppl. Fig. 2

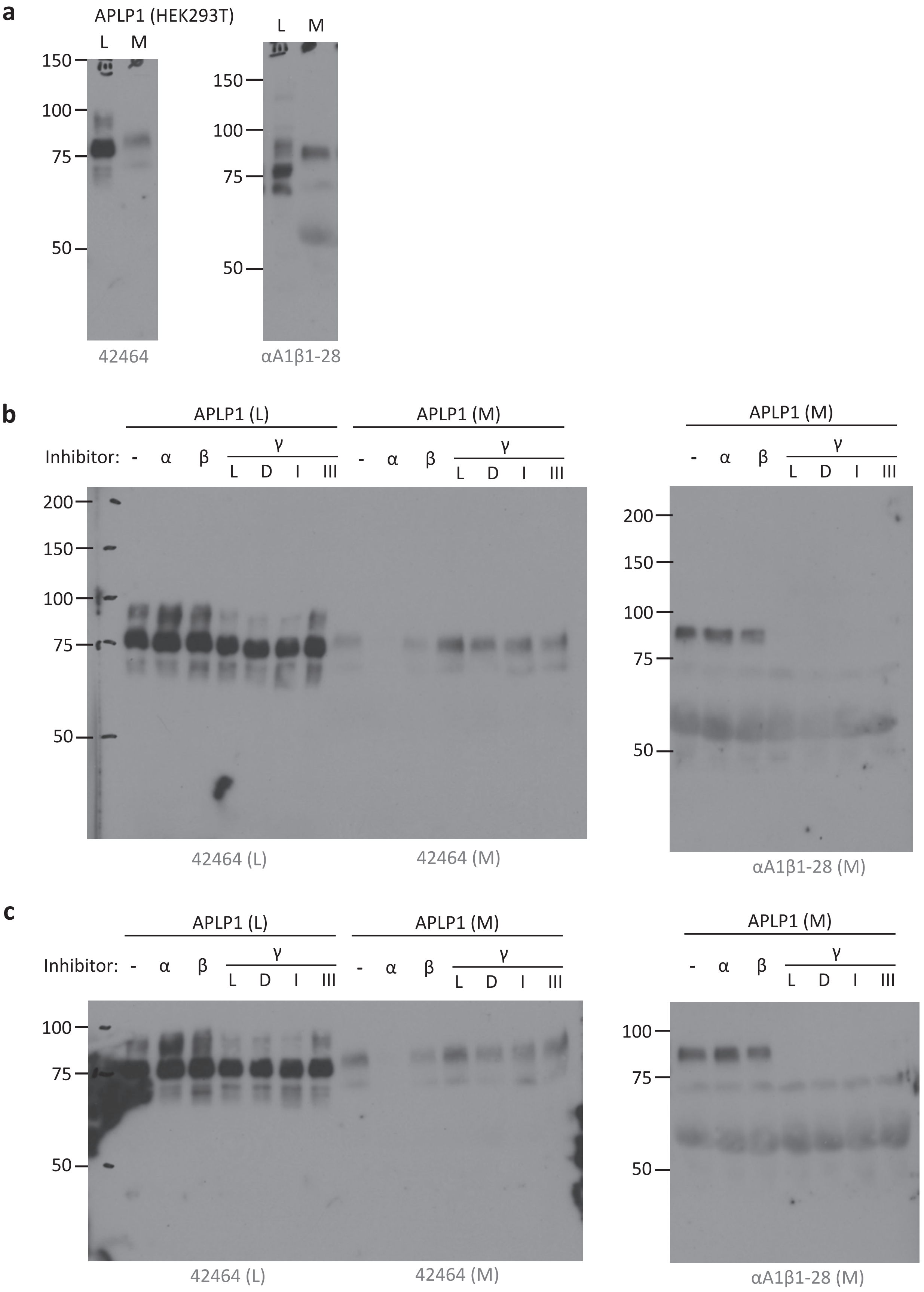
a
APLP1 (HEK293T)
L M
150
100
75
50
42464
L M
150
100
75
50
αA1β1-28
b
APLP1 (L)
APLP1 (M)
Inhibitor: - α β γ L D I III - α β γ L D I III
200
150
100
75
50
42464 (L)
42464 (M)
APLP1 (M)
- α β γ L D I III
200
150
100
75
50
αA1β1-28 (M)
c
APLP1 (L)
APLP1 (M)
Inhibitor: - α β γ L D I III - α β γ L D I III
100
75
50
42464 (L)
42464 (M)
APLP1 (M)
- α β γ L D I III
100
75
50
αA1β1-28 (M)

Suppl. Fig. 3

a

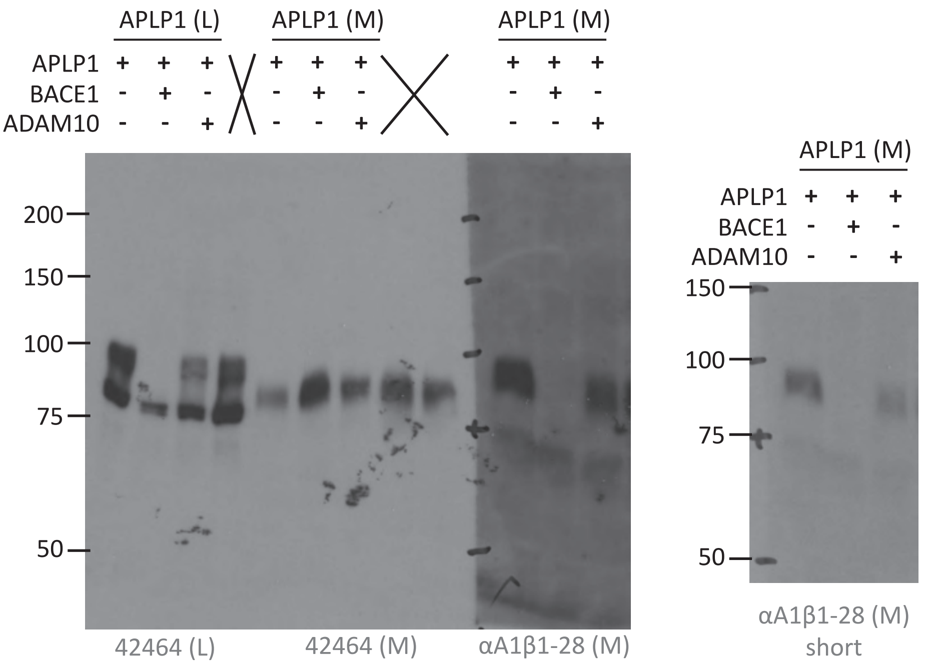

b

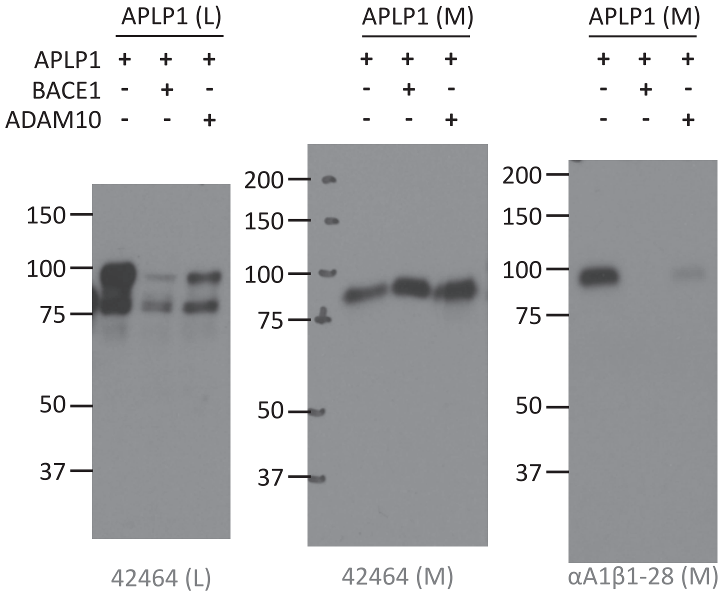

Suppl. Fig. 4

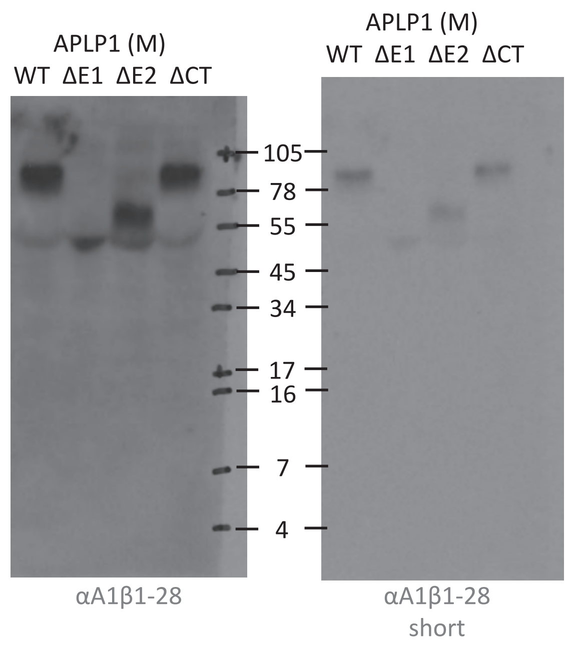
APLP1 (M)
WT ΔE1 ΔE2 ΔCT
105
78
55
45
34
17
16
7
4
αA1β1-28
APLP1 (M)
WT ΔE1 ΔE2 ΔCT
αA1β1-28
short

Suppl. Fig. 5

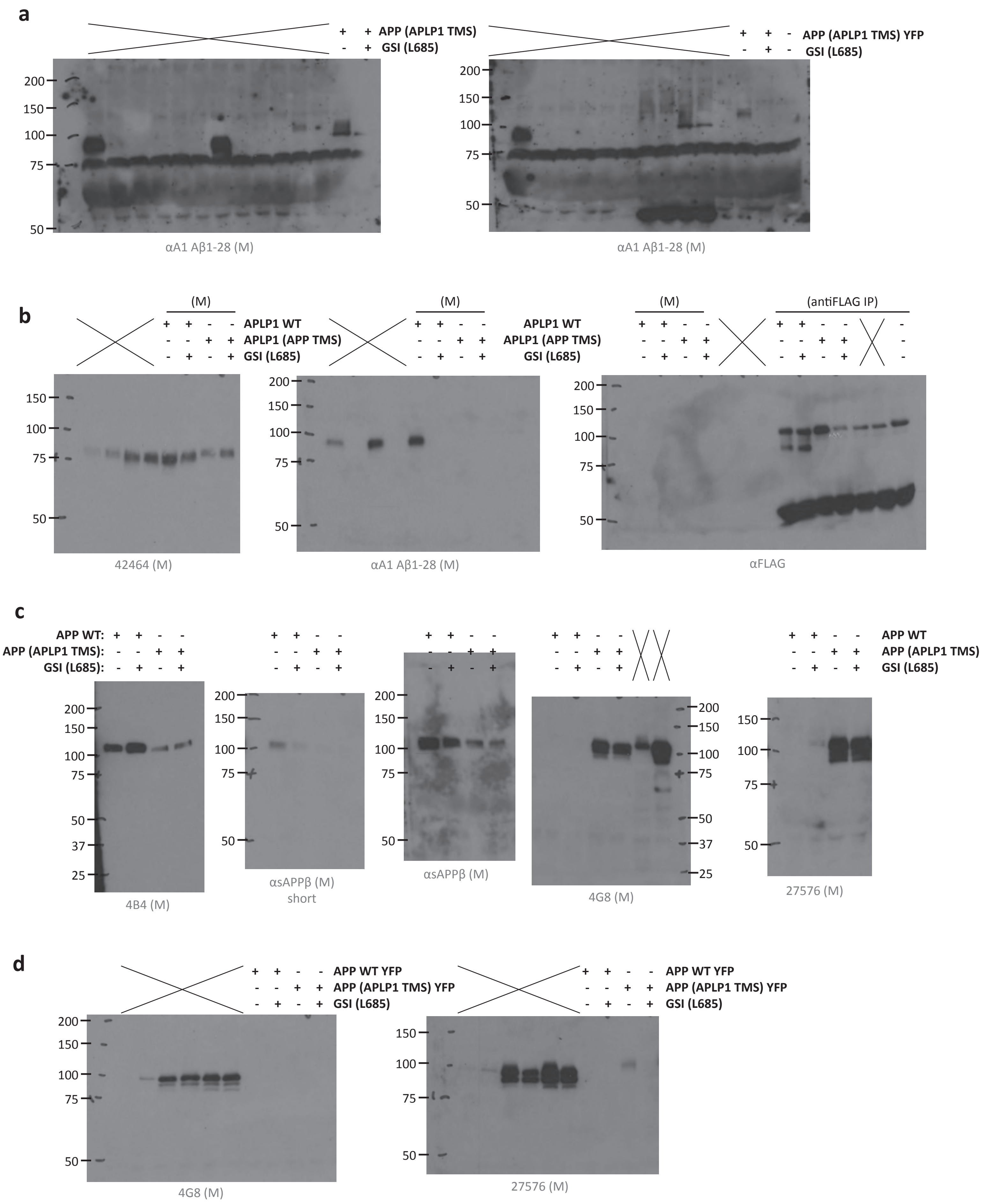
a
+ + APP (APLP1 TMS)
- + GSI (L685)
200
150
100
75
50
αA1 Aβ1-28 (M)
+ + - APP (APLP1 TMS) YFP
- + - GSI (L685)
200
150
100
75
50
αA1 Aβ1-28 (M)
b
(M)
+ + - - APLP1 WT
- - + + APLP1 (APP TMS)
- + - + GSI (L685)
150
100
75
50
42464 (M)
(M)
+ + - - APLP1 WT
- - + + APLP1 (APP TMS)
- + - + GSI (L685)
200
150
100
75
50
αA1 Aβ1-28 (M)
(M)
(antiFLAG IP)
200
150
100
75
50
αFLAG
c
APP WT: + + - -
APP (APLP1 TMS): - - + +
GSI (L685): - + - +
200
150
100
75
50
37
25
4B4 (M)
αsAPPβ (M)
short
αsAPPβ (M)
4G8 (M)
APP WT
APP (APLP1 TMS)
GSI (L685)
27576 (M)
d
+ + - - APP WT YFP
- - + + APP (APLP1 TMS) YFP
- + - + GSI (L685)
200
150
100
75
50
4G8 (M)
+ + - - APP WT YFP
- - + + APP (APLP1 TMS) YFP
- + - + GSI (L685)
150
100
75
50
27576 (M)
